# Complement Activation Contributes to the Pathophysiology of Shiga Toxin-Associated Hemolytic Uremic Syndrome

**DOI:** 10.3390/microorganisms7010015

**Published:** 2019-01-10

**Authors:** Simona Buelli, Carlamaria Zoja, Giuseppe Remuzzi, Marina Morigi

**Affiliations:** 1 Istituto di Ricerche Farmacologiche Mario Negri IRCCS, Centro Anna Maria Astori, Science and Technology Park Kilometro Rosso, 24126 Bergamo, Italy; simona.buelli@marionegri.it (S.B.); carlamaria.zoja@marionegri.it (C.Z.); giuseppe.remuzzi@marionegri.it (G.R.); 2 L. Sacco Department of Biomedical and Clinical Sciences, University of Milan, 20157 Milan, Italy

**Keywords:** Shiga toxin, hemolytic uremic syndrome, endothelial damage, microvascular thrombosis, podocytes, complement alternative pathway, long-term renal sequelae

## Abstract

Shiga toxin (Stx)-producing *Escherichia coli* (STEC) infections have become a threat to public health globally because of the severe illnesses that they can trigger, such as hemorrhagic colitis and the post-diarrheal hemolytic uremic syndrome (HUS), characterized by microangiopathic hemolytic anemia, thrombocytopenia, and acute kidney failure. Glomerular endothelial cells are primary targets of Stx which, after binding to its specific receptor globotriaosylceramide, upregulates proinflammatory proteins involved both in the recruitment and adhesion of leukocytes and thrombus formation at the site of endothelial injury. In this review, we discuss the role of complement activation in promoting glomerular microvascular dysfunction, providing evidence from experimental models and patients with STEC-HUS. Within the glomerulus, an important target for Stx-induced complement activation is the podocyte, a cell type that is in close contact with endothelial cells and participates in maintaining the filtration barrier. Recently, podocyte injury and loss have been indicated as potential risk factors for long-term renal sequelae in patients with STEC-HUS. Therapeutic approaches targeting the complement system, that may be useful options for patients with STEC-HUS, will also be discussed.

## 1. Introduction

Shiga toxin (Stx)-producing *Escherichia coli* (STEC) infections induce diarrhea, often epidemic, that can results in hemorrhagic colitis and hemolytic uremic syndrome (HUS), a disorder involving microangiopathic hemolytic anemia, thrombocytopenia, and acute renal failure, which develops mainly in early childhood [1,2,3,4,5]. HUS is categorized histopathologically as thrombotic microangiopathy, which consists of capillary wall thickening, swelling, endothelial cells detaching from the basement membrane, and fibrin- and platelet-rich thrombi obstructing the microvasculature of different organs, principally the kidney [6].

It has been estimated that STEC infections cause more than 2.8 million acute illnesses, 3890 cases of HUS, 270 cases of permanent end stage renal disease, and 230 deaths annually worldwide [7]. The overall incidence of STEC-HUS is about 2/100,000, reaching the peak of 6.1/100,000 in children under five years of age [2]. In Latin America, STEC infections are endemic, and Argentina has the highest incidence of the disease in the world (10–17 cases per 100,000 children under five years) [8]. The enterohemorrhagic *E. coli* (EHEC) O157:H7 serotype was responsible for most outbreaks around the world until 2010 [3,9]. Other STEC serotypes, including O26, O103, O111, O104, and O80, are now as frequent as O157:H7 in North America and Europe [3,9], but O157:H7 remains the predominant (>70%) serotype in Latin America [8]. In recent years, several Latin American groups have documented the emergence of new STEC serotypes and novel virulence factors in isolates from humans and animals, which indicates the genome plasticity of STEC strains and their capacity to evolve [10]. In 2011, an unusual *E. coli* O104:H4 caused a large outbreak of gastroenteritis and HUS in Germany that was associated with, but never conclusively proven to be due to ingestion of raw fenugreek sprouts [11]. This aggressive outbreak strain carried an unusual combination of the virulence loci characteristic to both enterohemorrhagic *E. coli*, a Stx-encoding bacteriophage chromosomally integrated, and enteroaggregative *E. coli*, a pAA plasmid-encoded aggregative adherence fimbriae promoting its tight adherence to epithelial cells [12,13,14,15]. The outbreak predominantly affected adults (89%), and caused the highest incidence rate of STEC-HUS ever recorded in the world, with exceptionally severe manifestations and high mortality rates [15,16].

STEC are commonly isolated from the gastrointestinal tracts of healthy ruminants, particularly cattle. STEC infection is most commonly acquired through ingestion of undercooked beef and unpasteurized milk, and cattle manure-contaminated vegetables and fruit or water [4,8]. A recent outbreak of STEC serogroups O121 and O26 in the United States was associated with contaminated flour from a large domestic producer [17]. A total of 56 cases were identified in 24 states. Thus, even though it is a low-moisture food, raw flour can transmit foodborne pathogens [17]. Cases of STEC infections through direct contact with infected animals after visits to farms or petting zoo or person-to-person transmission have been reported [4,8]. Some determinants of the pathogen, such as the serotype, horizontally acquired genetic elements (pathogenicity islands), the amount and type of Stx, as well as host factors, including age, immunity, lifestyle, use of antibiotics and antimotility agents, may influence the risk of acquiring a STEC infection [8,18,19].

Following STEC ingestion, illness manifests between 2 and 12 days later. The disorder begins with serious abdominal pain and non-bloody diarrhea associated with vomiting and fever and within 1–2 days can shift toward hemorrhagic colitis in 70% of cases. Disease progression into HUS occurs within a range of 3–9% of patients in sporadic cases, reaching 20% in some outbreaks. HUS is diagnosed 6 to 10 days after the onset of diarrhea, the time at which acute kidney failure starts. Acute mortality outcomes for patients with STEC-HUS have improved from 30% to less than 5% through the use of early dialysis [20]. However, of those patients who survive the initial insult, up to 25–30% develop renal sequelae or neurological manifestations [21,22].

## 2. Pathogenetic Mechanisms of STEC-HUS

After ingestion of contaminated food or water, many STEC colonize the intestinal mucosa producing a particular pathogenic process, the attaching/effacing (A/E) lesion, which is characterized by the disruption of microvilli, intimate attachment of the pathogen to the host enterocyte and alterations in the enterocyte cytoskeleton, with the creation of an actin-rich pedestal around the bacteria [23]. This promotes diarrhea and intestinal inflammation. Several important virulence/colonization factors have been described: the locus of the enterocyte effacement (LEE) pathogenicity island, responsible for the formation of the A/E lesions in the intestinal epithelium, encodes multiple effector molecules including a type III secretion system (T3SS), the adhesin intimin, and its translocated receptor Tir, which together with some host proteins are involved in actin polymerization and pedestal formation [24,25,26]. Conversely, several LEE-negative STEC were identified in the stool samples, indicating that the presence of LEE is not mandatory for bacterial infection in humans [27]. In fact, the German outbreak in 2011 has been triggered by the LEE-negative O104:H4 *E. coli* strain [27]. To successfully colonize the target organs, STEC can also produce different virulence factors as proteases that inactivate the complement system. This is the case for EspP, a serine protease autotransporter of the *Enterobacteriaceae* (SPATE) family, which cleaves and inactivates the complement cascade at C3 and C5 level, thus favoring bacterial survival in the host [28].

Stx released by STEC damages blood vessels in the colon, causing bloody diarrhea [1]. Then Stx passes through the gastrointestinal epithelium to enter the circulation and reach organs expressing its specific receptors (glycosphingolipid globotriaosylceramide [Gb3Cer] or globotetraosylceramide [Gb4Cer] receptors), such as the kidney, brain, and lungs [29,30]. Besides these glycolipids, Stx1 can bind to several molecules with higher affinity than Gb3Cer as glycans containing the amino sugar N-acetylglucosamine (GlcNAc), whereas Stx2 preferentially binds to the Pk mimic NAc-Galα1-4Galβ1-4Glc [31].

Lipopolysaccharide (LPS) is a component of the outer membrane of Gram-negative bacteria, which once developed in the gut by STEC may enter the systemic circulation and promote inflammation that contributes to damage in the Stx target organs. Old in vitro studies have shown that LPS potentiated Stx cytotoxicity on cultured endothelial cells by increasing the inhibitory activity of Stx on protein synthesis [32]. In an experimental model of HUS in baboons, LPS potentiated Stx toxicity through the upregulation of renal Stx receptors [33]. In mice, Stx together with LPS were required to induce a HUS-like response [34,35,36].

STEC may generate two major types of Stx: Stx1, Stx2 with different subtypes (Stx1a, Stx1c, Stx1d, Stx2a, Stx2b, Stx2c, Stx2d, Stx2e, Stx2f, and Stx2g) [37]. *E. coli* expressing Stx2 are responsible for more severe human disease. All Stxs have a common structure that includes one biologically active 32-kDa A subunit associated with five 7.7-kDa B subunits [38], which allow the toxin to bind to cells expressing Gb3Cer/CD77 or Gb4Cer receptors [39]. The toxin is endocytosed and undergoes retrograde transport to the Golgi apparatus and the endoplasmic reticulum [40]. Within the endoplasmic reticulum, the A subunit is proteolytically processed to form a 27 kDa A1-fragment, which is translocated into the cytosol and inactivates the ribosomes by removing an adenine residue from 28S ribosomal RNA, thereby interrupting protein synthesis and activating the ribosomal stress response [41]. Stx-induced ribotoxic stress activates multiple signaling pathways that may lead to proinflammatory (cytokines and chemokines) and apoptotic responses [24,42,43,44].

Negligible amounts of free Stx have been found in the sera of patients with HUS [45]; the detection of Stx2 may have been hampered by the presence in the human serum of Stx2-binding components, including serum amyloid P component. A new improved method may have overcome this problem, enabling early and sensitive detection of Stx2 in the sera of STEC-infected patients, so that preventive measures can be adopted in a timely manner [46]. Stx has been detected in the sera bound to blood cells or in blood cell-derived microvesicles. Erythrocytes, platelets, and monocytes are possible Stx carriers via the Gb3 receptor (for review see [47]). Human neutrophils do not express the Gb3 receptor but they interact with Stx through the Toll-like receptor 4 (TLR4), a protein receptor belonging to the pattern-recognition receptors of innate immunity [48]. Notably, TLR4 polymorphisms, which have been reported to affect the frequency and course of infectious diseases, could help to explain the individual susceptibility to STEC infections, causing mild or more serious clinical manifestations [48]. Microvesicles released by Stx-infected blood cells have been suggested as a mode of Stx transfer to glomerular cells [4,49]. Stx may be incorporated in blood microvesicles generated by neutrophils, monocytes, platelets, and erythrocytes, shuttled to the target cells and taken up after endocytosis of the entire microvesicle [49,50]. Thus, Stx may interact with its receptor (Gb3 or TLR4) on blood cells and be internalized within these cells [49] and once the toxin is shed from the blood cells within microvesicles, Stx can be taken up even by cells that lack Gb3 [50]. In addition to the toxin, microvesicles shed from platelets and monocytes may carry several other factors, such as activated complement components or tissue factors that can be transferred to the target cells [50].

## 3. Cytotoxic Effect of Shiga Toxin on Glomerular Endothelial Cells and Podocytes

**Figure 1:**
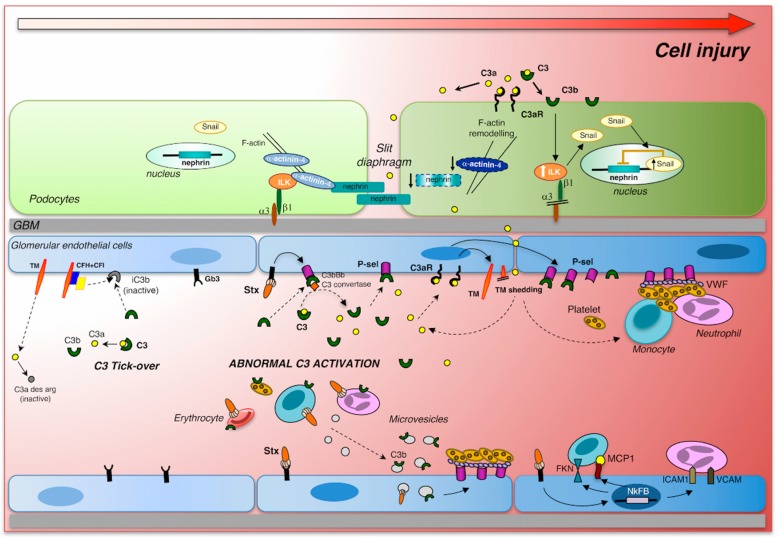
Intracellular signaling triggered by Shiga toxin (Stx) and complement activation in glomerular endothelial cells and podocytes. Stx binds to its specific Gb3 receptor and alters glomerular endothelial cell thromboresistance triggering a cascade of events that lead to microvascular thrombosis. Stx induces the release of thrombomodulin (TM) from the endothelial cell surface and upregulates the expression of P-selectin (P-sel), which interacts with von Willebrand Factor (VWF), thus promoting thrombus formation. Excessive glomerular complement activation and C3 deposition, in response to Stx, generates local C3a that, by binding C3a receptor, further enhances thrombomodulin shedding and P-selectin expression on endothelial cells, thereby favoring complement activation. In the systemic circulation, Stx can bind to neutrophils, monocytes, erythrocytes, and platelets, which in turn release microvescicles with surface-bound C3 that can contribute to the prothrombotic state in STEC-HUS. Stx, via NF-κB, directly induces the expression and production of MCP-1 and fractalkine (FKN), which, together with the adhesion molecules ICAM-1 and VCAM-1, favor leukocyte recruitment and adhesion to endothelial cells. In parallel, the C3a fragments in the systemic circulation can cross the glomerular filtration barrier or can be locally generated during C3 activation in the Bowman’s space in the proximity of podocytes. The binding of C3a to C3aR on the podocyte surface causes phenotypic changes including the reduction of α-actinin-4 expression and the increase of ILK-dependent nuclear translocation of Snail, with consequent nephrin downregulation and podocyte dysfunction and detachment. Adapted from Zoja et al., 2017 [63].

Glomerular endothelial damage has been indicated as one of the primary events in the development of the thrombotic microangiopathic lesions in STEC-HUS [24]. Shiga toxin induces profound alterations in endothelial cells, triggering a cascade of signaling events that result in the loss of endothelial anti-adhesive, anti-inflammatory, and thromboresistant properties (Figure 1). Shiga toxin, through the activation of the transcription factor nuclear factor-κB (NF-κB), enhances leukocyte adhesion to cultured human endothelial cells under flow conditions, by upregulating adhesive molecules (E-selectin, ICAM-1, and VCAM-1), and chemokines (MCP-1, IL-8, fractalkine) [36,51,52]. Gene expression profiling of human endothelial cells exposed to Stx documented the upregulation of proinflammatory cytokines, cell adhesion molecules, and NF-κB as well as the TNF/stress-related signaling pathways [53]. Stx mediates the loss of the thromboresistant phenotype of endothelial cells, triggering the formation of platelet thrombi on cultured human microvascular endothelial cells under high shear stress, which mimics that present in the microcirculation [54]. Von Willebrand factor (VWF), which undergoes conformational changes under high shear stress, was found to induce platelet adhesion on Stx-activated endothelial cells. Blockade of adhesive proteins, including P-selectin, decreased the formation of platelet thrombi on the endothelium [54]. In addition, it has been shown that Stx directly interacted with VWF, which caused a delay in the cleavage by the plasmatic metalloprotease ADAMTS13 of the VWF-platelet strings formed on activated endothelial cell surface [55]. Evidence that Stx induced the upregulation of endothelial chemokine receptor CXCR4 and increased plasmatic levels of its ligand stromal cell-derived factor-1 (SDF-1), indicated that the CXCR4/SDF-1 axis has an important role in Stx-induced endothelial activation. Indeed, treatment with CXCR4 antagonist limited thrombocytopenia and endothelial damage, and improved the survival of Stx-injected mice [56]. In an in vitro model of Stx-induced glomerular endothelial cell injury, incubation with endothelial progenitor cells (EPC) in co-culture resulted in a regenerative effect in terms of increased glomerular endothelial cell viability, which was associated with the release of the growth factors VEGF, IGF-1, FGF-2, and HGF in the supernatant [57]. These findings suggest that treatment strategies applying EPC or EPC-released growth factors could be possibly used in the clinic.

Within the glomerulus, the podocytes, which are in close proximity to endothelial cells and participate in the glomerular filtration barrier, are also susceptible to the cytotoxic effects of Stx [58,59,60,61]. Upon binding to the Gb3 receptors, Stxs activated human podocytes to release cytokines, such as IL-1 and TNF-α which, through the upregulation of Gb3 expression, enhanced sensitivity toward the toxin [59,62] and favored apoptosis [61]. In cultured mouse podocytes, Stx2 activated p38 and p42/44 mitogen-activated protein kinases (MAPKs), and upregulated the transcription factors NF-κB and AP-1 [58], important regulators of cytokine and chemokine gene expression. Stx caused a marked rearrangement of the podocyte cytoskeleton through the production of the vasoactive peptide endothelin-1 (ET-1), implying that Stx may alter glomerular hemodynamics via an autocrine and paracrine action of podocyte-derived ET-1 [58].

## 4. The Complement System

A large body of evidence collected over the last three decades shows that complement activation contributes to the pathophysiology of STEC-HUS [63,64,65,66]. The complement system was discovered approximately 100 years ago, as a group of heat-sensitive plasmatic proteins that enhance the opsonization and elimination of bacteria by antibodies with a function complementary to that of humoral immunity. Nowadays, complement should instead be considered as a system that links several responses during immune and inflammatory reactions, and not merely as a bacterial killer [67].

The complement system comprises over 30 components, including membrane-bound regulators, receptors, and numerous plasma proteins. Complement (C) is activated through the classical, lectin, or alternative pathways [68]. The activation of the classical pathway starts by the initial binding of C1q to antibodies generated during the humoral response or by inflammatory proteins, including C-reactive and serum amyloid proteins. The lectin pathway initiates without the presence of antibodies with the recognition of certain oligosaccharide moieties on the pathogen surface by mannose-binding lectin (MBL) proteins. The alternative pathway is initiated by the spontaneous hydrolysis of C3, leading to the formation of C3(H_2_O) and the binding of a small amount of C3b to carbohydrates and proteins on the cell surface. All three routes converge in the generation of C3b by the C3 convertases; however, the fate of C3b deposits determines whether the cell surface will be destroyed or not. If the initial C3b deposits are not rapidly inactivated, then complement cascade continues and C3b participates in further C3 convertase formation, followed by the formation of the membrane attack complex (MAC, C5b-9) [68].

To prevent over-activity of the pathways and to limit the deposition of complement proteins on host cells, the complement cascade is finely regulated by the presence of a number of fluid phase (complement factor H and factor I) and membrane-bound (CD55, CD46, and CD59) regulatory proteins that promote the cleavage of C3b to the inactive form iC3b, the dissociation of C3/C5 convertases, and prevent C9 assembly into the C5b-9 complex [69].

## 5. Stx-associated HUS and Complement Activation: Clinical and Experimental Evidence

First reports from the literature documented reduced C3 and augmented serum levels of the complement breakdown products C3b, C3c, and C3d in children during the active phase of the disease [70,71,72]. Low levels of C4 have occasionally been observed in patients with STEC-HUS [73]. Complement activation occurred via the alternative pathway as factor Bb plasma levels were increased in the serum of 17 children studied during the onset of the disease [74]. Recent data have confirmed the presence of high plasma levels of factor Bb and soluble C5b-9, a sensitive indicator of overall complement activation, in patients with active STEC-HUS and demonstrated a correlation between these parameters and oliguria [75]. Signs of complement activation in the acute phase of the disease were also found in a cohort of 10 children with elevated plasma levels of C3a, generated by the cleavage of C3 into C3b and sC5b-9, which returned to normal after recovery [73]. In addition to the systemic activation of complement, intrarenal C3 and C5b-9 deposits, together with fibrin accumulation, were found in the glomeruli of a STEC-HUS child, suggesting a functional link between complement activation and renal microvascular thrombosis [76].

In vitro evidence indicates that Stx directly regulates the activation of the complement system. Stx2 activates complement in the fluid phase, leading to the formation of soluble C5b-9 when added to normal human serum [77]. Stx can interact with complement proteins and activate the alternative pathway. Direct binding of Stx2 to factor H, the major soluble inhibitor of the alternative pathway, specifically at the short consensus repeat (SCR) 6–8 and 18–20, the regions responsible for host surface recognition, was observed by Orth and colleagues [77]. Stx2 binding impaired factor H cell surface cofactor activity, resulting in increased complement activation and C3b accumulation, while factor H fluid phase regulation was maintained [77]. Other studies have shown that Stx2 is also a ligand for two other proteins belonging to the factor H family, namely FHR-1 and FHL-1, which show amino acid sequence and regulatory function similarities to factor H [78]. Moreover, Stx2 modulates the expression of CD59, a membrane-bound complement regulator which inhibits the formation of the C5b-9 complex. Lower surface expression of CD59 was found on human glomerular endothelial cells exposed to Stx2 due to a reduction of CD59 mRNA [79]. Further evidence that complement has a role in the pathogenesis of STEC-HUS comes from the observation of C3 and C9 deposits on the surface of blood cell microparticles in patients with STEC-HUS [73,80]. The exposure of whole blood to Stx2 caused the formation of platelet-leukocyte aggregates with surface-bound C3 and C9 [73]. Deposition of complement-activated products on platelets led to the release of microparticles that may contribute to the prothrombotic state in STEC-HUS [81]. Moreover, Stx2 promoted the release of hemoglobin and the formation of red blood cell-derived microvesicles coated with C3 and C5b-9 [80]. The release of microvescicles induced by Stx2 was inhibited in the absence of factor B, suggesting that complement, via the alternative pathway, is involved in the hemolytic process that occurs in STEC-HUS [80].

A number of animal models have provided important information about the key role of the alternative pathway of complement activation in the thrombotic microangiopathic lesions leading to kidney dysfunction in STEC-HUS. Deposits of C3 and C5b-9 were found in the glomeruli of mice infected with STEC [76]. Early treatment with anti-C5 antibody and C6 deficiency prevented renal disease progression [76]. Mice with HUS induced by coinjection of Stx2 plus LPS showed thrombocytopenia and renal function impairment, paralleled by glomerular C3 deposition and fibrin(ogen) accumulation [36,82,83]. The hypothesis that the alternative complement pathway is a key mediator for microvascular thrombosis is based on data demonstrating protection from platelet loss [82] and renal dysfunction [83] in mice with factor B deficiency. Abnormal activation of the alternative pathway can also be achieved through the activation of factor B and D by MBL/ficolin-associated serine proteases (MASPs), indicating the possibility that there is an indirect way of complement activation [84]. Recent evidence suggests the lectin pathway has a role in disease onset, since the inhibition of MBL2 in mice with Stx-HUS remarkably limited renal C3d deposition and renal injury [85].

## 6. Complement Activation Induces Glomerular Endothelial Damage

The overactivation of the complement system, as occurs in Stx-HUS, significantly undermines renal vascular function, leading to the acquisition of a prothrombotic state (Figure 1). It is well established that Stx1 and Stx2 directly cause complement activation, via the alternative pathway, on the surface of endothelial cells. In vitro experiments have shown that following perfusion with whole blood, microvascular endothelial cells pre-treated with Stx1 exhibit increased C3 deposition and a larger cell surface covered by thrombi than cells pre-exposed to control medium [82]. Similar results were obtained with Stx2. Proof that C3 deposition is functionally connected to thrombus growth rests on the finding that thrombus formation on the endothelial surface was completely inhibited by the complement inhibitor soluble complement receptor 1 (sCR1). Endothelial complement deposition due to Stx1 is mediated by the upregulation of the membrane adhesive molecule P-selectin, which has been shown to bind C3b with high affinity and to activate the alternative pathway [82,86]. P-selectin blockade limited Stx-induced complement activation and thrombus formation on perfused endothelial cells in vitro [82]. Moreover, treatment with anti P-selectin antibody protected mice with Stx-HUS against glomerular endothelial damage and thrombosis [82].

Endothelial dysfunction induced by Stx1 and Stx2 is characterized by loss of thrombomodulin, a transmembrane glycoprotein receptor for thrombin, known to regulate endothelial thromboresistance [87]. In addition to its involvement in coagulation, thrombomodulin possesses properties that impact on fibrinolysis, complement activation, inflammation, and cell proliferation [88]. In Stx-HUS mice, the lack of the lectin-like domain of thrombomodulin caused defective complement regulation and increased their susceptibility to developing thrombocytopenia and renal dysfunction [87]. In vitro, Stx1 reduced thrombomodulin expression in endothelial cells by directly promoting their shedding from the cell surface through the release of serine proteases from endothelial Weibel-Palade bodies [82] (Figure 1). In mice with Stx-HUS, glomerular expression of thrombomodulin was reduced, and this was associated with C3 fibrin(ogen) and platelet thrombi [82]. These experimental findings support the clinical evidence that genetic or acquired functional defects in thrombomodulin may favor the adverse outcomes during STEC-HUS. Missense mutations that alter thrombomodulin function, leading to defective complement regulation, have been identified in patients with atypical HUS, a form that is not STEC-associated [88]. A recent study provides evidence for a causal link between microvascular thrombosis and complement activation demonstrating that the interaction between the thrombogenic multimeric VWF and C3 and C5b-9 deposits leads to shedding of thrombomodulin on activated endothelial cells exposed to sera from patients with congenital thrombotic microangiopathy [89].

Complement-activated proteins, such as C3a, C5a, and C5b-9, carry out proinflammatory activities and promote the perturbation of physiological endothelial thromboresistance through several synergic mechanisms. First, both C3a and C5a binding to their receptors, as well as the deposition of sublytic amounts of C5b-9, induced the upregulation of adhesion molecules and cytokine secretion, which increased cell permeability and promoted leukocyte adhesion on endothelial cells [90,91,92,93]. Moreover, C3a and C5a possess potent chemotactic and activating properties, which can exacerbate the accumulation of primed leukocytes on damaged endothelium [94]. C3a, C5a and sublytic C5b-9 also directly induced platelet activation by depolarizing the membrane potential, thereby causing granule secretion and the release of procoagulant microparticles [81,95,96]. On the other hand, these complement-activated proteins act on endothelial cells by promoting the loss of anticoagulant surface heparan sulfate proteoglycans, causing cytoskeletal rearrangement, with consequent cell retraction and exposure of the procoagulant extracellular matrix [97,98]. In response to C3a, activated endothelial cells exhibited remarkable increased P-selectin expression and thrombomodulin loss, which translated in platelet thrombus formation on the endothelial surface [82]. The pathogenic role of C3a in microvascular thrombosis in Stx-HUS has been highlighted by data demonstrating that treatment with a C3a receptor antagonist markedly reduced fibrin(ogen) deposits and limited thrombomodulin loss in the glomeruli of Stx-HUS mice [82].

## 7. Complement Activation Induces Glomerular Podocyte Injury

Over 25–30% of STEC-HUS patients who do not fully recover from the acute disease experience long-term renal sequelae with a critical reduction in nephron numbers and consequent hyperfiltration, proteinuria and chronic kidney disease (CKD) [21,22,99]. Clinical findings from the one- or five-year follow-up have indicated persistent proteinuria as a poor prognostic factor for progressive CKD [100,101,102]. The general consensus from experimental and human studies recognizes glomerular podocyte injury and podocyte loss as precipitating events in the complex processes leading to glomerular diseases associated with proteinuria [103,104,105,106]. Podocytes are post-mitotic cells that are unable to proliferate and to replenish their numbers following migration or detachment from the glomerular basement membrane during kidney injury, whatever the primary kidney disease [107,108]. In STEC-HUS, little information is available regarding the impact of glomerular podocyte injury on the onset of proteinuria, because kidney biopsies are rarely performed. However, the characteristic lesions of thrombotic microangiopathy associated with the collapse and retraction of the glomerular tuft can be accompanied by a swelling of podocytes and foot process effacement [71,102,109]. Evidence that mRNAs for nephrin and synaptopodin, two podocyte-specific proteins, were found in the urine of 15 children with active STEC-HUS is a convincing indication that podocyte damage and loss occurred in these patients [110], making this a potential biomarker of long-term renal outcomes. These findings are supported by experimental evidence from a baboon model of HUS, in which glomerular endothelial injury was functionally linked to structural podocyte changes [111]. Furthermore, rats injected with Stx2 developed microalbuminuria, which can be considered an early sign of podocyte injury, as demonstrated by the concomitant altered glomerular pattern of nephrin and podocalyxin expression [112]. A recent study has shown that mice infected with STEC exhibited increased levels of platelet- and leukocyte-derived microvescicles coated with complement proteins in the renal microcirculation, from where they were transferred to glomerular endothelial cells and podocytes, possibly favoring cell injury [49,73].

While searching for important determinants of glomerular injury that may be predictive of long-term renal prognosis in HUS, our group has demonstrated that complement activation plays a crucial role in podocyte damage in experimental HUS [83] (Figure 1). In mice with Stx2/LPS-induced HUS, glomerular deposition of C3 was accompanied by podocyte dysfunction and loss [83]. In these animals, glomerular complement accumulation was shown to activate critical regulators of podocyte adhesion, migration, and cell–cell interaction, such as integrin-linked kinase (ILK) signaling, as well as the transcription factor Snail, which is responsible for the downregulation of nephrin [83]. Moreover, when applying the Stx2/LPS model to factor B-deficient mice, complete recovery of glomerular architecture was observed, clearly indicating that the activation of complement via the alternative pathway promotes podocyte dysfunction [83]. Interestingly, an unrecognized role that intraglomerular C3a plays, as the direct effector of glomerular podocyte damage, was discovered. The evidence that treatment with a C3a receptor antagonist was renoprotective and limited podocyte depletion in HUS mice [83] further indicates that C3a is a possible new therapeutic target for patients with STEC-HUS. The clear proof-of-concept that C3a directly affects podocyte phenotype is based on in vitro data showing that the activation of ILK and its downstream effector, Snail, increased motility and migration of human podocytes in response to C3a by altering the expression of the cytoskeletal protein α-actinin 4 [83]. Consistent with these findings, the pathogenic role of C3a/C3a receptor in the dysregulation of podocytes has been confirmed in another model of progressive nephropathy [113]. Thus, aberrant glomerular C3 deposition and C3a generation triggered podocyte injury, and subsequently activated parietal epithelial cells, leading to the development of glomerulosclerotic lesions in mice with protein overload proteinuria [113]. Altogether, the above findings are relevant from a clinical perspective, because targeting C3a, instead of C3, can avoid the adverse effects of increased susceptibility to infection due to the complete shutdown of C3 activity.

## 8. Treatments

The general management of STEC-HUS patients includes correctly monitoring electrolyte and water imbalance, anemia, hypertension, and renal failure. To limit the severity of acute kidney injury and the need for renal replacement therapy, the administration of intravenous fluids and sodium, as soon as a STEC infection is suspected, may be required. In contrast, plasma exchange and immunoabsorption have been administered in complicated cases, in spite of the paucity of evidence confirming their efficacy [9,63,114].

Though increasing evidence indicates there is heightened complement activation in STEC-HUS patients, with the generation of C3b, Bb, C3a, sC5b-9, and C9 in the circulation or deposited on platelet-leukocyte aggregates and microvescicles [49,73,80], the precise underlying pathogenic mechanism of tissue damage remains unclear. Clinical studies have demonstrated the beneficial effects of the complement inhibitor eculizumab, a humanized monoclonal anti-C5 antibody, in patients with atypical HUS as well as paroxysmal nocturnal hemoglobinuria, myocardial infarction, age-related maculopathy, and C3 glomerulopathy [115]. Given these premises, the efficacy of eculizumab has been tested in patients with STEC-HUS who are deteriorating, in order to halt the progression of the disease [116,117]. The first case series was described by Lapeyraque et al. [118] in which three young children with STEC-HUS with severe neurological involvement, were treated with eculizumab, which resulted in dramatic improvements in their neurological symptoms and hematological parameters. These impressive data represented the rationale for raising awareness of the potential of treating patients with severe HUS with eculizumab and were the reason why clinicians administered eculizumab during the devastating German outbreak that started in May 2011, and involved over 4000 people, of whom among 850 progressed to HUS [119,120,121]. It has been reported that the disease outcome did not seem to be affected appreciably by the use of eculizumab combined with plasmapheresis, compared to plasmapheresis alone [119,120,121]. However, these findings could be interpreted in a completely different way. Outcomes were similar between groups, but those given plasmapheresis had more severe diseases. Despite the central nervous system involvement, the patients on eculizumab tended to have less major complications [122]. Thus, data from the above studies are inconclusive, as they are strongly biased by the retrospective and nonrandomized design of the studies. More recent reports, ranging from case series to cohort studies, provide varied results that seem to indicate that eculizumab treatment, particularly in patients with severe neurological dysfunction, has beneficial effects if given early [116]. Overall, the current data in patients with STEC-HUS neither definitively confirm nor disprove the efficacy of eculizumab. Two ongoing, double-blinded placebo-controlled clinical trials—ECULISHU in France (NCT02205541), which is focusing on renal disease, and ECUSTEC in the UK (ISRCTN89553116), which is addressing general disease severity—are attempting to provide convincing evidence to help manage and support the use of eculizumab in STEC-HUS patients [117].

## 9. Conclusions

A growing body of evidence, as detailed in this review, highlights the role of the virulence factor Stx as the prerequisite for the activation of the complement via the alternative pathway in the pathophysiology of STEC-HUS (Figure 1). Glomerular endothelium has long been considered to be the main target of Stx-induced renal cytotoxicity. However, recent studies point to glomerular podocytes as a cell population—which is in close proximity to injured glomerular endothelial cells—that is heavily involved in disease progression. Complement activation and the generation of C3a in response to Stx, trigger a series of events that affect the glomerulus, starting with the loss of endothelial thromboresistance and development of microvascular thrombi. These changes are accompanied by podocyte dysfunction and loss, which could be a risk factor for the long-term renal sequelae that occur in 25–30% of STEC-HUS patients. Experimental evidence suggests that complement blockade at the C3 and C3a level could be a possible therapeutic option for counteracting glomerular injury triggered by Stx. So far, the only complement inhibitor used in clinical practice has been the anti-C5 antibody eculizumab, but results obtained in STEC-HUS patients have been controversial. Looking to the future, new therapeutics are entering the clinic [115]. They target different proteins of the complement system, have different half-lives, different ways of administration, each has its own range of activities and side effects. This could be encouraging for the treatment of several diseases, including STEC-HUS.
